# Gallbladder Adenomyomatosis Presenting With Abdominal Pain

**DOI:** 10.7759/cureus.10485

**Published:** 2020-09-16

**Authors:** Shravan Teelucksingh, Tonya Welch, Adrian Chan, Jason Diljohn, Fidel S Rampersad

**Affiliations:** 1 Department of Radiology, The University of the West Indies, Port of Spain, TTO

**Keywords:** gallbladder adenomyomatosis, mri, ct

## Abstract

A previously well 50-year-old male presented with a six-year history of worsening right-sided upper abdominal pain, postprandial nausea, and early satiety. His blood tests, including full blood count, liver biochemistry, and serum amylase, were normal. CT of the abdomen with intravenous contrast demonstrated concentric segmental mural thickening of the body and fundus of the gallbladder, with intramural cystic foci (rosary sign). MRI of the abdomen demonstrated segmental gallbladder mural thickening with fluid-filled intramural diverticula (pearl necklace sign) and an hourglass configuration of the gallbladder, consistent with segmental gallbladder adenomyomatosis. The patient subsequently underwent laparoscopic cholecystectomy with histological confirmation of gallbladder adenomyomatosis, without evidence of malignancy. His postoperative recovery was uneventful.

## Introduction

Gallbladder adenomyomatosis is a relatively common entity, characterized by gallbladder epithelial proliferation and mural muscular hypertrophy [1], with resultant gallbladder wall thickening. Additionally, multiple mucosal outpouchings develop into or through the thickened gallbladder wall, referred to as the Rokitansky-Aschoff sinuses. Sonographically evident gallbladder wall thickening usually has its etiology in inflammatory or neoplastic conditions, with cholecystitis and gallbladder carcinoma being the two major causes. Utilizing CT and MRI, the presence of diffuse gallbladder wall thickening with intramural diverticula forming the “rosary sign” and “pearl necklace sign” and an hourglass configuration of the gallbladder usually clinch the radiological diagnosis of gallbladder adenomyomatosis [2]. Herein we report a case of symptomatic gallbladder adenomyomatosis that demonstrated the classical radiological features and was subsequently confirmed histologically upon laparoscopic cholecystectomy.

## Case presentation

A 50-year-old male presented to the accident and emergency department with a six-year history of worsening right-sided upper abdominal pain, nausea, and early satiety. There was no history of fatty food intolerance, fever, or vomiting. There was also no weight loss, jaundice, dark urine, or pale stools. His physical examination was unremarkable, with no abdominal tenderness or palpable mass. 

Routine blood investigations were ordered (full blood count, liver function tests, and serum amylase), and were found to be normal. Abdominal ultrasound revealed mild gallbladder wall thickening, no evidence of cholelithiasis, and no pericholecystic fluid (images not available). 

**Figure 1:**
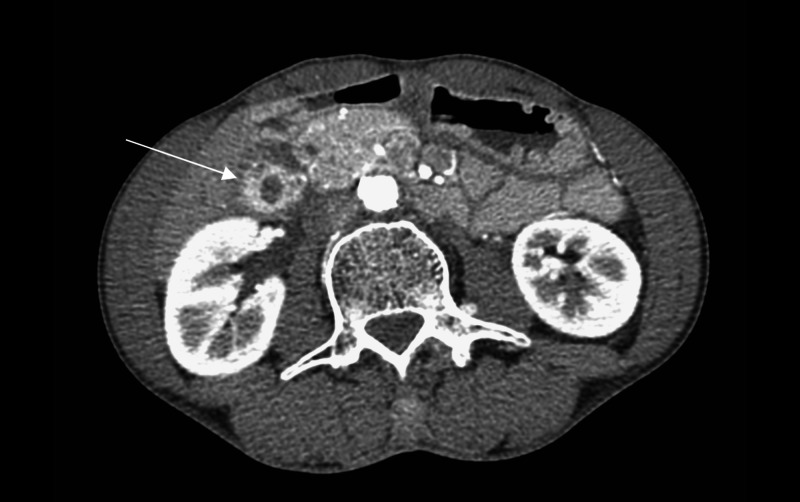
Axial contrast-enhanced CT image shows concentric thickening and enhancement of the gallbladder wall at the body and fundus with small mural cystic foci (arrow) representing the “rosary sign”.

Intravenous contrast-enhanced CT of the abdomen showed segmental concentric thickening and enhancement of the gallbladder wall, confined to the body and fundus, with tiny intramural cystic foci (rosary sign). There was no dominant gallbladder mass, pericholecystic fat stranding, pericholecystic fluid, or hyperdense calculi. The intrahepatic and extrahepatic ducts were normal. There was no regional lymphadenopathy (porta hepatis and coeliac axis) (Figure 1). 

**Figure 2:**
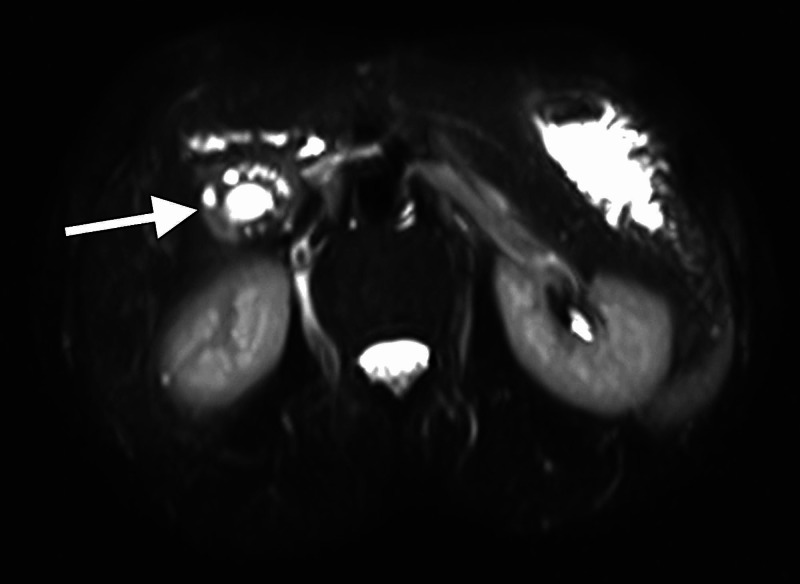
Axial T2-weighted MRI image shows mural thickening, fluid-filled intramural diverticula (arrow) representing the “pearl necklace sign”.

**Figure 3:**
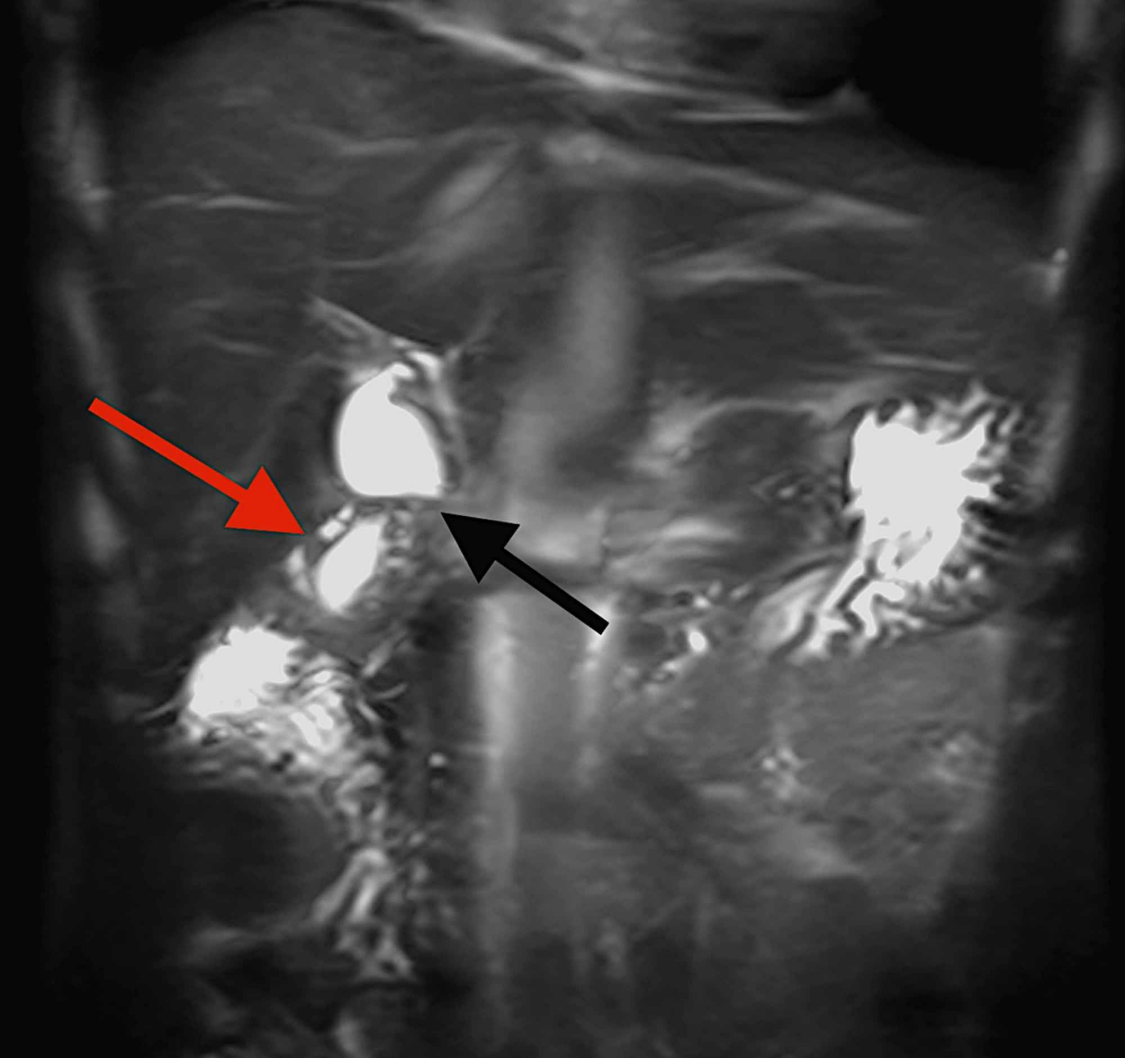
Coronal T2-weighted MRI image showing mural thickening of the gallbladder fundus and body with fluid-filled intramural diverticula (red arrow) and an hourglass configuration (black arrow) consistent with gallbladder adenomyomatosis (segmental type).

After general surgery review, an MRI of the abdomen was requested. The MRI also demonstrated segmental gallbladder mural thickening, with fluid-filled intramural diverticula (pearl necklace sign) (Figure 2). The gallbladder assumed an hourglass configuration (Figure 3), with normal pericholecystic fat. There was no dominant mass to suggest a neoplasm. No choleliths or choledocholiths were identified, nor were there any acute inflammatory changes in the gallbladder, liver, or pancreas. The radiological features were thus consistent with gallbladder adenomyomatosis (segmental type).

**Figure 4:**
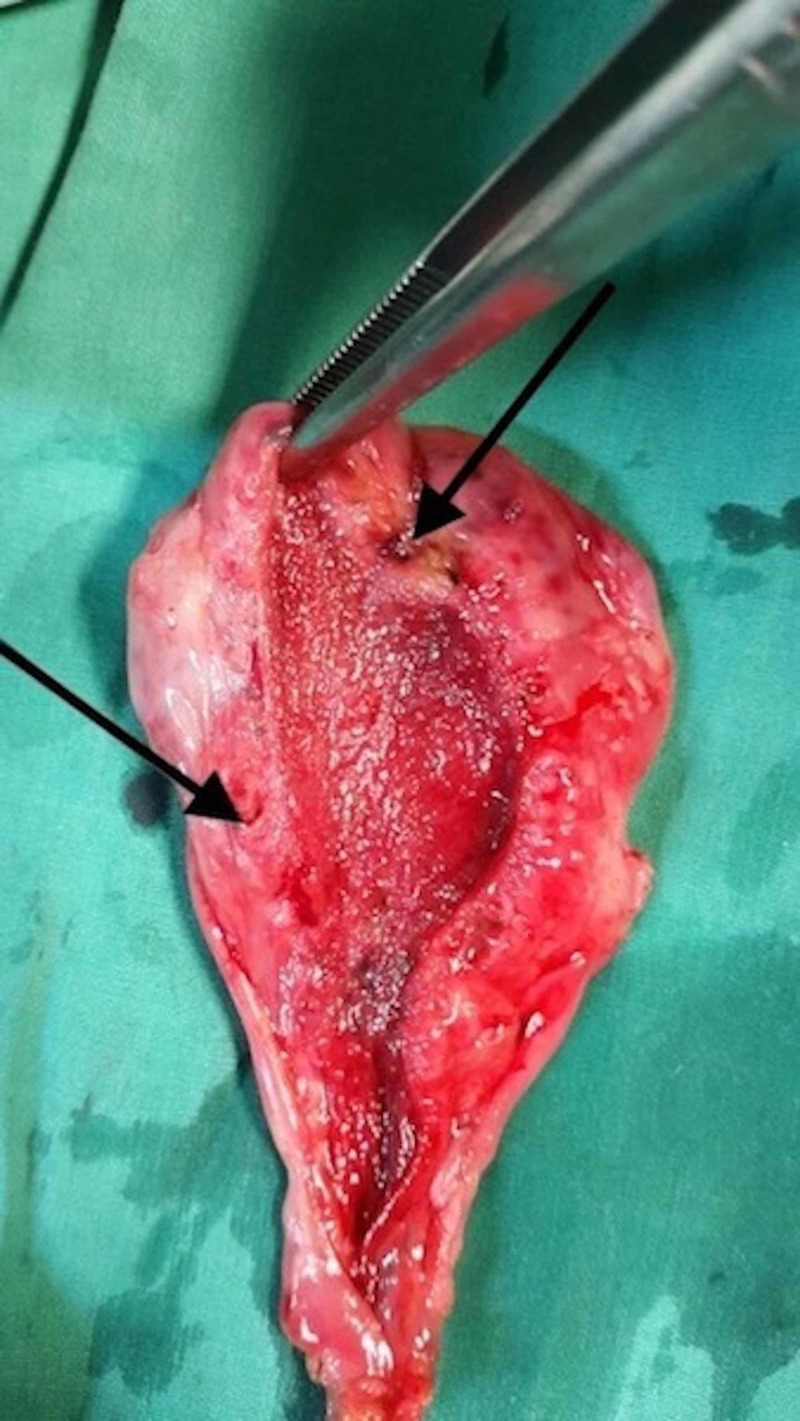
Image of a longitudinal gross section of the gallbladder shows thickening of the gallbladder wall at the body and fundus and multiple cystic intramural cavities (arrows) corresponding to Rokitansky-Aschoff sinuses.

**Figure 5:**
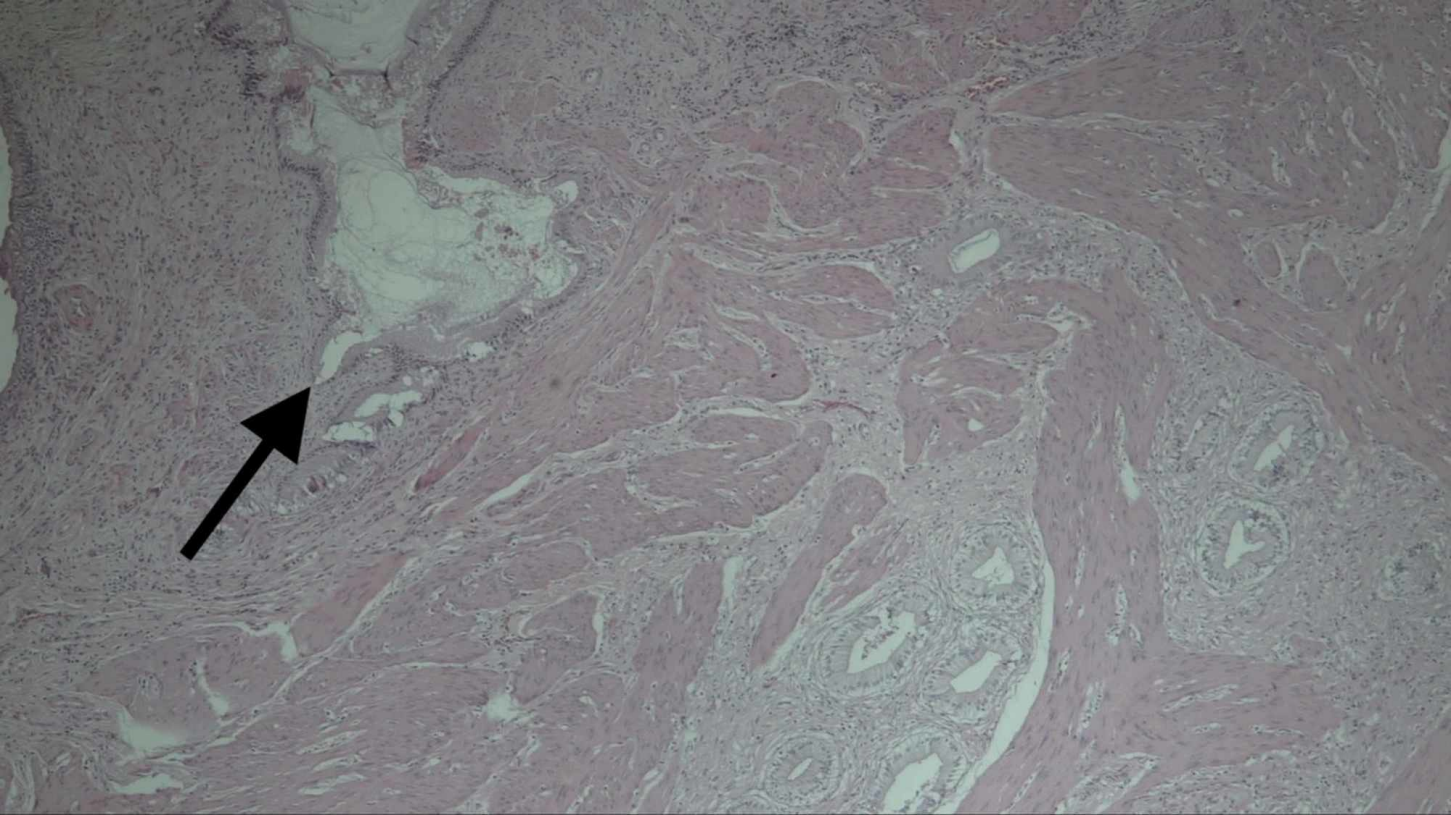
Micrograph with H and E stain showing (black arrow) herniation of mucosa into muscular wall (Rokitansky-Aschoff sinus) and other cystic glandular formations with a thickened gallbladder wall, consistent with gallbladder adenomyomatosis.

The patient was offered a laparoscopic cholecystectomy. This was performed with no intraprocedural challenges or complications. Pathologic and histologic examinations confirmed gallbladder adenomyomatosis (Figures 4, 5). There was an uneventful postoperative period with early discharge and outpatient follow-up. The patient indicated resolution of the symptoms on follow-up visit. 

## Discussion

Gallbladder wall thickening has many etiologies, the most commonly encountered being cholecystitis, adenomyomatosis, and gallbladder cancer [3]. Gallbladder adenomyomatosis is characterized by focal or diffuse gallbladder mural thickening with invagination of the epithelium, forming Rokitansky-Aschoff sinuses [4]. Muscle hypertrophy, impaired gallbladder drainage, and increased pressure in the gallbladder lumen result in Rokitansky-Aschoff sinuses formation [5]. 

Gallbladder adenomyomatosis is a relatively common condition, which is found in 1%-9% of all cholecystectomy specimens [6]. Four patterns of gallbladder adenomyomatosis are described: localized, segmental, fundal, and diffuse [7]. Our patient had the segmental type, involving the gallbladder body and fundus, which separated the gallbladder into two compartments [8,9], giving it an hourglass configuration.

The condition is usually asymptomatic and may be discovered incidentally during radiological imaging [10,11]. Adenomyomatosis of the gallbladder can however manifest with abdominal pain [4,12], presenting with intermittent bouts of right upper quadrant pain similar to symptomatic gallbladder disease (biliary colic and cholecystitis) [13,14]. Nausea and vomiting associated with meals and fatty food intolerance are also reported symptoms. 

The diagnosis can sometimes be made on ultrasound, when there are intramural cystic foci and "comet tail" artifacts. This is more sensitive with high-resolution ultrasound scanning, but is operator dependent. MRI demonstrates gallbladder wall thickening and the characteristic "pearl necklace" appearance due to intramural cystic spaces called Rokitansky-Aschoff sinuses [15]. MRI is particularly useful in equivocal cases to help differentiate adenomyomatosis from gallbladder carcinoma (accuracy of 93%) [6]. CT has been considered somewhat limited for the detection and differentiation of adenomyomatosis from gallbladder carcinoma; however, the diagnosis of adenomyomatosis can be made with reasonable accuracy when thickening of the gallbladder wall is seen to contain small cystic-appearing spaces, as in this case [4]. The small mural cysts seen on CT have been referred to as the “rosary sign”.

Symptomatic adenomyomatosis is considered an indication for cholecystectomy, while asymptomatic disease is not an indication for surgery. If there is any clinical or radiological doubt about the possibility of adenocarcinoma of the gallbladder, a cholecystectomy is usually warranted [7]. Gallbladder adenomyomatosis is also considered a premalignant condition [14], with a higher incidence of gallbladder malignancy from the segmental type [16].

## Conclusions

This case highlights the value of CT and MRI for the evaluation of gallbladder mural thickening in the era of multimodality imaging. The identification of intramural cystic foci on CT and MRI representing Rokitansky-Aschoff sinuses is essential for the diagnosis of adenomyomatosis of the gallbladder. CT and MRI can help differentiate cholecystitis or gallbladder adenocarcinoma from adenomyosis. Additionally, segmental gallbladder mural thickening giving the hourglass configuration and a presentation of right upper quadrant abdominal pain should raise the possibility of symptomatic gallbladder adenomyomatosis, thus preventing delays in management. 
